# Feasibility of using a novel multibending accessory device designed for a slim therapeutic endoscope

**DOI:** 10.1055/a-2689-3462

**Published:** 2025-09-11

**Authors:** Tomohiro Shimada, Taku Yamagata, Yoshihide Kanno, Takeshi Shimizu, Kai Korekawa, Hiroki Sato, Kei Ito

**Affiliations:** 1 Department of Gastroenterology, Sendai City Medical Center, Sendai, Japan

A multibending scope is useful in gastric endoscopic submucosal dissection (ESD) for early gastric cancer (EGC), especially in anatomically challenging areas, such as the fornix and the anterior wall of the gastric body, where close access is often difficult using conventional therapeutic endoscopes 1 2 . However, use of the multibending scope is restricted because it is produced by a single company and available only in a limited number of institutions.

To overcome this limitation, a novel dedicated disposable accessory, the AttachBend (Fujifilm Medical Co., Tokyo, Japan), was developed for the EG-840TP slim therapeutic endoscope. This is an external assistive deflection device mounted on the endoscope to provide additional multibending functionality. Although the device has an outer diameter of 13.5 mm, it does not compromise the slim profile of the scope’s 7.9-mm distal tip. It enables one-way angulation of up to 70 degrees, and by rotating the attachment, deflection can be directed in the desired direction ( Fig. 1 ).

**Fig. 1:**
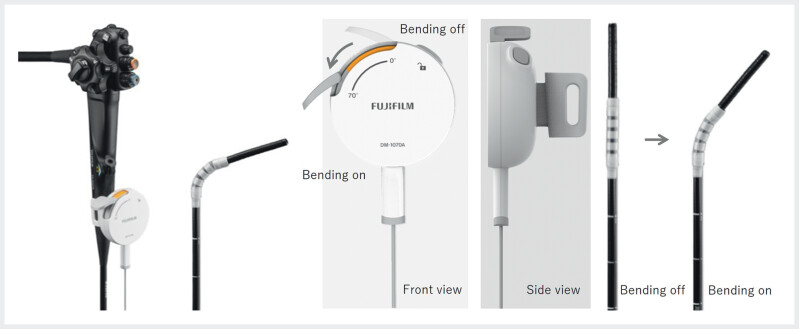
The AttachBend is an external assistive deflection device (outer diameter, 13.5 mm) designed exclusively for the EG-840TP. It enables one-way angulation of up to 70 degrees at a second bending segment located 10 cm from the tip. The direction of deflection can be adjusted by rotating the attachment, which is controlled by a lightweight manual lever mounted below the working channel.

We present the case of an 83-year-old man who underwent ESD using the EG-840TP for an 85-mm EGC located along the lesser curvature of the gastric body ( Video 1 **,** Fig. 2 ). As the dissection advanced toward the anterior wall, close approach became anatomically challenging. The use of a novel multibending accessory allowed adequate proximity to this challenging area, providing optimal working distance and angulation to the lesion and enabling effective continuation of the dissection.

Feasibility of using a novel multibending accessory device designed for a slim therapeutic endoscope.Video 1

**Fig. 2:**
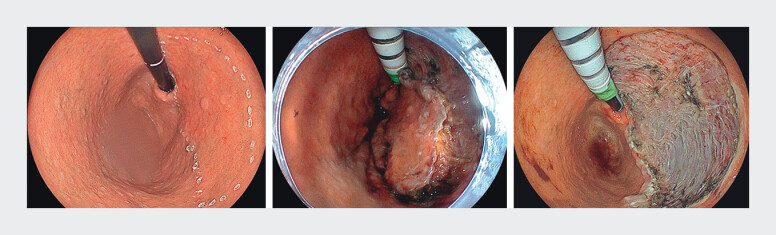
An 85-mm early gastric cancer was located along the lesser curvature of the gastric body, limiting access. The use of a novel multibending accessory facilitated approach to the lesion, allowing endoscopic submucosal dissection to be performed at an appropriate working distance.

Even in facilities without access to the multibending scope, a novel multibending accessory, which can be easily mounted onto the EG-840TP, might offer a practical and versatile alternative, simulating the functionality of the multibending scope for anatomically difficult gastric lesions.

Endoscopy_UCTN_Code_TTT_1AO_2AG_3AC

## Notes

Endoscopy E-Videos https://eref.thieme.de/e-videos
*E-Videos* is an open access online section of the journal *Endoscopy* , reporting on interesting cases and new techniques in gastroenterological endoscopy. All papers include a high-quality video and are published with a Creative Commons CC-BY license. Endoscopy E-Videos qualify for HINARI discounts and waivers and eligibility is automatically checked during the submission process. We grant 100% waivers to articles whose corresponding authors are based in Group A countries and 50% waivers to those who are based in Group B countries as classified by Research4Life (see: https://www.research4life.org/access/eligibility/ ). This section has its own submission website at https://mc.manuscriptcentral.com/e-videos .
